# A Tb (Ⅲ) Coordination Polymer Based on 5-(2-(Pyrazole-1-yl) Pyridine-5-yl) Terephthalic Acid and Its Visual Detection of Quinolone Antibiotics

**DOI:** 10.3390/polym17172277

**Published:** 2025-08-22

**Authors:** Ai Wang, Yichong Li, Wei Zhao, Jia Liu

**Affiliations:** 1 Key Laboratory of Chemical Biology and Molecular Engineering of the Education Ministry, Institute of Molecular Science, Shanxi University, Taiyuan 030006, China; liyichong1010@163.com (Y.L.); zw18435740421@163.com (W.Z.); liujia18635024993@163.com (J.L.); 2 Key Laboratory of Materials for Energy Conversion and Storage of Shanxi Province, Shanxi University, Taiyuan 030006, China

**Keywords:** coordination polymer, fluorescence detection probe, quinolone antibiotics, visual detection, fluorescence sensing mechanism

## Abstract

The abuse of quinolone antibiotics in the medical and livestock industries potentially causes environmental accumulation that may impair ecological stability. Based on the organic ligand 5-(pyrazole-1-yl) pyridine-5-yl) terephthalic acid (H_2_PPIPA), a terbium(III) complex, [Tb(HPPIPA)(PPIPA)(H_2_O)]ₙ (complex **1**), was synthesized via solvothermal reaction with Tb(NO_3_)_3_·6H_2_O. Luminescence studies revealed that complex **1** functions as a turn-on fluorescent probe for the selective detection of ofloxacin (OFX), levofloxacin (LFX), and norfloxacin (NFX), with detection limits of 27.9, 17.1, and 8.0 nM, respectively. Owing to its high selectivity and anti-interference capability, the complex was successfully applied for the determination of OFX and LFX in milk samples. Furthermore, a test strip impregnated with complex **1** enabled naked-eye fluorescence detection of OFX, LFX, and NFX under 254 nm UV light. Additionally, a fluorescence sensing film fabricated from complex **1** exhibited excellent recyclability, allowing for at least seven consecutive detection cycles without significant signal loss. This study innovatively designed and synthesized a novel Tb(III)-based coordination polymer fluorescent probe utilizing an original ligand scaffold, achieving the first reported visual detection of quinolone antibiotics with fluorescence test strips and agar films.

## 1. Introduction

Quinolone antibiotics, as a class of broad-spectrum antimicrobial agents, exhibit potent therapeutic efficacy against infections caused by both Gram-positive and Gram-negative bacteria [1,2]. However, their indiscriminate use can lead to bacterial resistance, organ toxicity, and residue accumulation in animal-derived food products (e.g., milk, eggs, meat, and honey). These residues may bioaccumulate in humans, posing significant health risks [3,4]. Consequently, the monitoring and detection of quinolone antibiotics in environmental samples and food matrices are of critical importance. Currently, common analytical methods for the determination of these compounds include high-performance liquid chromatography (HPLC), colorimetric analysis, capillary electrophoresis (CE), and enzyme-linked immunosorbent assay (ELISA) [5,6,7,8]. However, these conventional techniques suffer from several limitations, such as high instrumentation costs, moderate sensitivity, and the inability to achieve real-time or visual detection [9,10,11]. Consequently, the development of a reliable, efficient, and rapid detection method for quinolone antibiotics has garnered increasing research interest.

In recent years, lanthanide metal–organic frameworks (Ln-MOFs) have emerged as versatile platforms for antibiotic detection due to their exceptional luminescent properties. Compared with conventional transition metal–organic complexes, the Tb(III) and Eu(III) complexes—with their fingerprint-like green (^5^D_4_→^7^F_5_ transition at 545 nm) and red emissions (^5^D_0_→^7^F_2_ transition at 615 nm), respectively—are particularly prevalent in sensing applications owing to their large Stokes shifts (> 200 nm) and microsecond-scale lifetimes [12,13,14]. For instance, a novel Eu-MOF [15] has been developed for ratiometric fluorescence detection of quinolone antibiotics and selective sensing of tetracycline antibiotics, demonstrating successful application in monitoring antibiotic residues in lake water. Additionally, two new three-dimensional Ln-MOFs [16] based on Eu(III)/Tb(III) cations were synthesized, which function as highly efficient quenching sensors for detecting norfloxacin (NFX) and ciprofloxacin (CIP) in urine samples. Nevertheless, existing coordination polymer-based probes cannot concurrently satisfy the requirements of ultra-low LODs and real-time visual identification for quinolone antibiotics.

Herein, a novel lanthanide metal–organic complex **1** [Tb(HPPIPA)(PPIPA)H_2_O]_n_ was synthesized successfully by using Tb (III) ions and 5-(2-(pyrazole-1-yl) pyridine-5-yl) terephthalic acid (H_2_PPIPA). The ligand incorporates diverse N- and O-donor atoms in its architecture, where the multi-typic coordination sites (as opposed to single-binding-site ligands) enable the construction of coordination complexes with enhanced structural diversity and unique physicochemical properties. Furthermore, the simultaneous presence of pyrazole and pyridine rings in the ligand scaffold facilitates metal coordination through stable five-membered chelate formation, demonstrating superior metallophilicity compared to mono-heterocyclic analogs, thereby significantly reducing synthetic challenges.

The new complex exhibits intense green luminescence under 254 nm UV irradiation, demonstrating characteristic Tb^3+^ emission in its fluorescence spectrum. Remarkably, the characteristic emission peaks of Tb^3+^ in complex **1** showed significant enhancement upon addition of ofloxacin (OFX), levofloxacin (LFX), and norfloxacin (NFX), suggesting its potential as a turn-on fluorescent probe for detecting these quinolone antibiotics. To evaluate its practical application, we investigated the sensing performance of complex **1** for quinolone antibiotics in milk samples. Furthermore, we fabricated a fluorescent agarose membrane incorporating complex **1**, which enables rapid, convenient, and visual identification of OFX, LFX, and NFX through naked-eye observation under UV light.

## 2. Experimental Section

### 2.1. Synthesis of [Tb(HPPIPA)(PPIPA)H_2_O]_n_ (Complex ***1***)

A reaction mixture containing Tb(NO_3_)_3_·6H_2_O (0.2 mmol, 90.6 mg), and H_2_PPIPA (0.2 mmol, 63.2 mg) in a mixed solvent system (CH_3_CN/H_2_O, 2:6 *v*/*v*, 8 mL total volume) with 100 μL HNO_3_ (1 M) was sealed in a 15 mL PTFE-lined stainless steel autoclave. The reaction vessel was heated to 413 K under autogenous pressure and maintained at this temperature for 72 h. After slow cooling to room temperature automatically, colorless block crystals of the target complex were obtained. The product was isolated by filtration, washed repeatedly with distilled water, and air-dried, yielding 57% (based on H_2_PPIPA).

### 2.2. X-Ray Crystal Diffraction Data of Complex ***1***

Single-crystal X-ray diffraction data for complex **1** were collected at 273(2) K on a Bruker D8 QUEST diffractometer equipped with a PHOTON 200 CMOS detector and Mo-*Kα* radiation (λ = 0.71073 Å), employing a graphite monochromator. Absorption corrections based on equivalent reflections were applied using the program SADABS [17]. The structure was solved by intrinsic phasing methods using OLEX2 [18] and refined by full-matrix least-squares techniques on *F*^2^ with SHELXL-2018 [19]. All non-hydrogen atoms were refined anisotropically. Hydrogen atoms were placed in calculated positions and refined using a riding model. Complete crystallographic data, including refinement parameters and convergence results, have been deposited with the Cambridge Crystallographic Data Centre (CCDC no. 2416360). Detailed crystallographic information and refinement statistics are provided in Table S1 (Supplementary Materials).

### 2.3. Fluorescence Detection Experiment

A total of 5 mg of crystalline complex **1** was ground into a fine powder and dispersed in 50 mL of distilled water, followed by ultrasonication for 30 min. After allowing the suspension to stand undisturbed for 3 days, a homogeneous and stable supernatant was obtained for fluorescence measurements. For spectral analysis, 2 mL of the supernatant was transferred to a quartz cuvette to record the baseline fluorescence emission spectra. To evaluate the sensing performance, aliquots (1 × 10^−3^ M) of the following analytes were individually introduced into the supernatant:

Ofloxacin (OFX), norfloxacin (NFX), levofloxacin (LFX), tetracycline (TC), chlortetracycline (CTC), oxytetracycline (OTC), ornidazole (ORN), trimethoprim (TMP), sulfamethoxazole (SMZ), ibuprofen (IBU), gemfibrozil (GEM), diclofenac (DCF), and p-acetylaminophenol (APAP).

### 2.4. Test Strip Preparation

The preparation protocol involved dispersing 10 mg of complex **1** powder in 50 mL of distilled water via 30 min ultrasonication, followed by 3-day sedimentation to obtain a stable suspension. After being filtered by a 2 μm filter, filter paper with a size of 5 × 0.5 cm^2^ was soaked in the filtrate of complex **1** for 1 h, and the filter paper strip was taken out and placed in an oven at 70 °C to dry for 1 h.

### 2.5. Fluorescent Sensing Film

A total of 200 mg of agar powder and 50 mg of complex **1** were homogenized in 10 mL of distilled water within a 25 mL beaker. The mixture was heated at 90 °C for 15 min to ensure complete dissolution, after which 1 mL of the filtrate was cast into a cylindrical mold (radius = 1 cm, height = 0.25 cm) and allowed to solidify for 1 h at room temperature.

## 3. Results and Discussion

### 3.1. Crystal Structure of Complex ***1***

**Figure 1:**
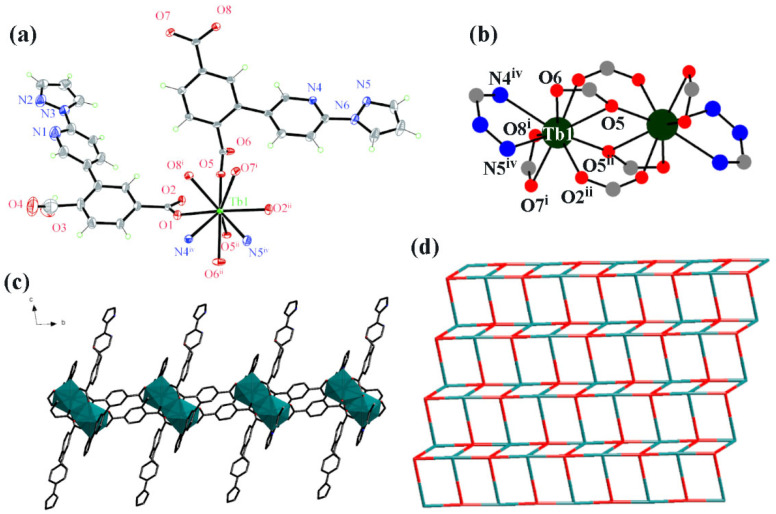
(**a**) Coordination environment of complex **1** (30% ellipsoid degree, symmetry codes: (i) −x + 1, −y + 1, −z + 1; (ii) −x + 1, −y, −z + 1; (iii) x − 1, y, z; (iv) x + 1, y, z); (**b**) Di-nuclear structure of complex **1**; (**c**) One dimensional chain of complex **1**; (**d**) Topological network diagram of complex **1**. Color codes: blue—Tb^3+^ node; red—[HPPIPA]^−^/[PPIPA]^2−^ node.

Complex **1** crystallized in the triclinic system with space group Pī. The asymmetric unit of **1** comprises one Tb^3+^ ion, one fully deprotonated PPIPA^2−^ ligand, one partially deprotonated HPPIPA^−^ ligand, and one crystalline water molecule. As shown in Figure 1a (30% probability ellipsoids), the Tb(III) center exhibits a nine-coordinate [TbO_7_N_2_] geometry, coordinated by O1, O2^ii^ O5, O5^ii^, O6^ii^, O7^i^, and O8^i^ [symmetrical codes: (i) −x + 1, −y + 1, +1 − z; (ii) −x + 1, − y − z + 1; (iii) x − 1, y, z; (iv) x + 1, y, z] from the two-part deprotonated HPPIPA^−^ and three fully deprotonated PPIPA^2−^. Two adjacent Tb(III) ions are connected by four COO- groups from four different ligands, leading to a dinuclear secondary building unit (SBU) (Figure 1b). These dinuclear units propagate along the *b*-axis through fully deprotonated carboxylate bridges, generating 1D polymeric chains. In the *ac* plane, the ligands interconnect Tb(III) centers through both pyridyl-N and pyrazolyl-N donors, assembling the 1D chains into a corrugated 2D network with stair-step topology (Figure 1c). And Figure 1d illustrates the two-dimensional topological network architecture of complex **1** with the Schläfli symbol {3^2^·5^8^}·{3^6^}. The phase purity was confirmed by PXRD, where the experimental pattern matches well with the simulated pattern from single-crystal data (Figure S1 in the Supplementary Materials).

### 3.2. Fluorescence Detection of Quinolone Antibiotics

**Figure 2:**
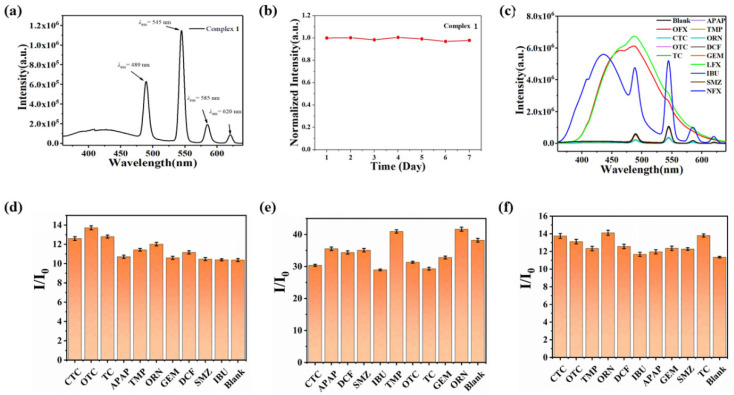
(**a**) Liquid fluorescence of complex **1**; (**b**) Fluorescence intensity of complex **1** in water over 7 days; (**c**) Fluorescence intensity of complex **1** after adding different antibiotics; (**d**–**f**) Anti-interference experimental diagram of complex **1** for detection of (**d**) NFX, (**e**) LFX, and (**f**) OFX towards other antibiotics.

Upon excitation at 316 nm, complex **1** shows four characteristic Tb^3+^ emission bands at 489 nm, 545 nm, 585 nm, and 620 nm, which can be assigned to the energy transfer of ^5^D_4_→^7^F_6_, ^5^D_4_→^7^F_5_, ^5^D_4_→^7^F_4,_ and ^5^D_4_→^7^F_3_, respectively [20,21]. The dominant ^5^D_4_→^7^F_5_ transition at 545 nm confers bright green luminescence to the complex, as visually demonstrated in Figure 2a. And photostability assessment (Figure 2b) confirms remarkable aqueous stability of complex **1**, with negligible fluorescence attenuation (ΔI < 5%) after continuous monitoring for 7 days. Therefore, based on the excellent fluorescence emission stability of complex **1**, the detection performance of complex **1** in the environment will be explored as follows.

Upon the addition of 200 μL aliquots of various antibiotics (1 × 10^−3^ M) to 2 mL of complex **1**, only ofloxacin (OFX), levofloxacin (LFX), and norfloxacin (NFX) induced a significant enhancement in the fluorescence emission at 489 nm (Figure 2c). Notably, NFX also produced a pronounced increase in emission intensity at 545 nm. Given that OFX and LFX exhibit intrinsic fluorescence at 489 nm in aqueous solution, further investigations into the sensing selectivity of complex **1** were conducted by monitoring the 545 nm emission band, which is exclusively attributed to the Tb^3+^ center (^5^D_4_→^7^F_5_ transition).

To evaluate the selective detection capability of complex **1**, we investigated its anti-interference performance against OFX, LFX, and NFX in the presence of competing antibiotics. As shown in Figure 2d–f, the characteristic emission peak at 545 nm (corresponding to the ^5^D_4_→^7^F_5_ transition of Tb^3+^) exhibited significant enhancement upon addition of OFX, LFX, or NFX, even when coexisting with other antibiotics. These results demonstrate that the fluorescence detection performance of complex **1** remains unaffected by potential interferents, confirming its excellent selectivity for quinolone antibiotics.

**Figure 3:**
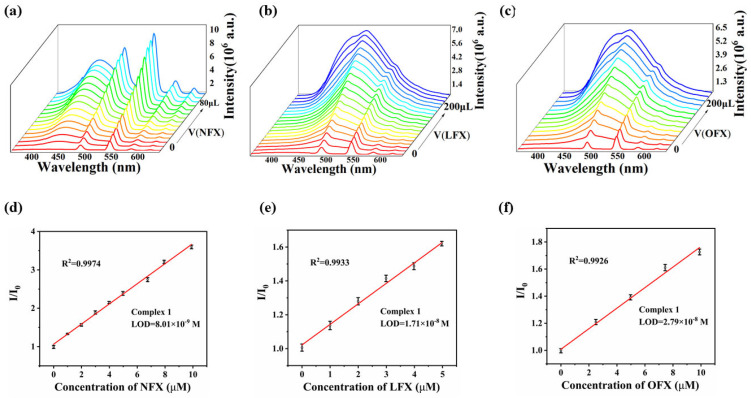
The change of fluorescence spectra of complex **1** with the increase in the concentration of (**a**) NFX, (**b**) LFX, and (**c**) OFX and the linear relationship between complex **1** and low concentrations of (**d**) NFX, (**e**) LFX, (**f**) OFX in an aqueous solution.

To further investigate the sensing performance of complex **1**, fluorescence titration experiments were conducted by progressively introducing OFX, LFX, and NFX into the supernatant of complex **1**. As illustrated in Figure 3a–f, a linear relationship between the fluorescence intensity *I*/*I*_0_ and the concentrations of NFX and OFX was observed, and the detection limit (LOD) is 8.01 nM for NFX and 27.9 nM for OFX by using the formula LOD = 3*σ*/*S* (where *σ* represents the relative standard deviation of the fluorescence intensity of five blank solutions, and *S* is the slope of the linear curve). There is a good linear relationship between fluorescence intensity *I*/*I*_0_ and LFX concentration in the range of 0–5 μM. Using the formula LOD = 3*σ*/S, the detection limit (LOD) is 17.1 nM. These results demonstrate that complex **1** serves as a highly sensitive fluorescent probe for detecting quinolone antibiotics (OFX, LFX, and NFX) in aqueous media. Compared with the currently reported fluorescence sensors, their detection limits are low (see Table S3).

### 3.3. Detection of OFX and LFX in Pretreated Commercial Milk

Initially, 3 mL of commercial whole milk was diluted 20-fold with distilled water in a 50 mL centrifuge tube. Subsequently, 15 mL of 2% (*w*/*v*) trichloroacetic acid (TCA) was added for protein precipitation, followed by 15 min of ultrasonication. The deproteinized solution was centrifuged at 6000 rpm for 10 min, and the supernatant was filtered through a 0.22 μm membrane to remove residual lipids [22,23,24]. The filtrate was then adjusted to pH 7 using PBS buffer (matching the pH stability of complex **1** suspension shown in Figure S3 of the Supplementary Materials).

Known concentrations of target analytes (OFX and LFX) were spiked into the pretreated milk samples, followed by 30 min of ultrasonication for complete homogenization. As demonstrated in Table 1, the method validation results showed the recovery rates of standard OFX are 98.93% ~ 106.73%, with the relative standard deviation (RSD, n = 3) < 3.1%; for LFX, the satisfactory recovery rates are 97.07%~104.87% with RSD (n = 3). These analytical performance characteristics confirm that complex **1** serves as a reliable fluorescence sensor for the quantitative determination of OFX and LFX residues in milk samples, meeting international validation criteria for food safety analysis.

### 3.4. Visual Detection of Quinolone Antibiotics

**Figure 4:**
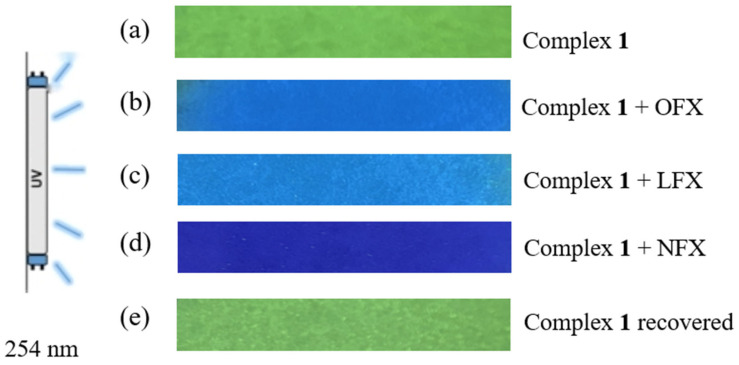
Under the irradiation of 254 nm ultraviolet lamp, (**a**) Filter paper strips soaked in aqueous suspension of complex **1**; (**b**) Filter paper strips with OFX dripped on the surface; (**c**) Filter paper strips with LFX dripped on the surface; (**d**) Filter paper strips with NFX dripped on the surface; (**e**) Filter paper strips washed with distilled water.

To evaluate the practical application of complex **1** for on-site detection, we developed a fluorescent test strip [25,26,27]. The prepared filter paper with a size of 5 × 0.5 cm^2^ was soaked in the filtrate of complex **1** for 1 h. And the filter paper strip was taken out and placed in an oven at 70 °C to dry for 1 h. Then, under the irradiation of a 254 nm ultraviolet lamp, as shown in Figure 4a, the test strip is obviously green. Then, by adding (b) OFX, (c) LFX, and (d) NFX with the concentration of 1 × 10^−3^ M, respectively, the strips showed a distinct colorimetric transition to blue. Remarkably, the original green emission was restored after eluting the antibiotics with distilled water (Figure 4e), demonstrating the strips’ reversibility and reusability.

**Figure 5:**
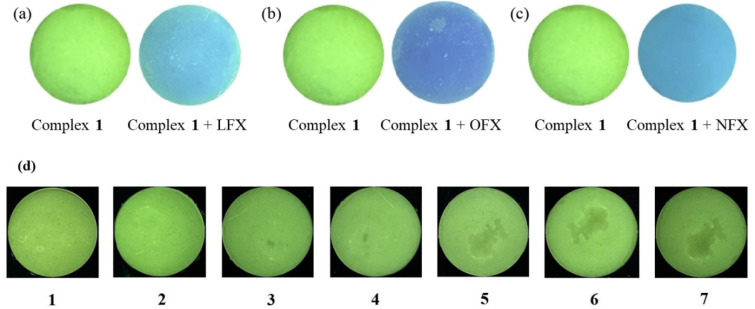
Under a 245 nm ultraviolet lamp, the color changes of fluorescence sensing films before and after adding (**a**) LFX, (**b**) OFX, and (**c**) NFX; (**d**) Recycling performance of the fluorescent sensing membrane.

Furthermore, agar films containing complex **1** are applied to visually detect the three quinolone antibiotics. Then, upon exposure to quinolone antibiotics (OFX, LFX, and NFX), the film exhibited distinct colorimetric transitions under UV light (Figure 5a–c). Notably, the film demonstrated excellent reversibility, with the original fluorescence signal fully restored after seven consecutive detection–washing cycles using distilled water (Figure 5d). These results confirm that the agar-embedded complex **1** film serves as a robust, reusable platform for the visual detection of quinolone antibiotics.

### 3.5. Mechanism of Detection

**Figure 6:**
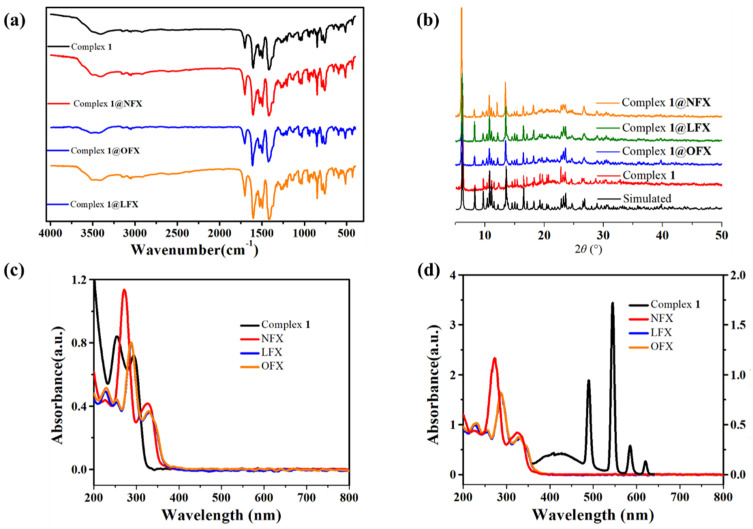
(**a**) Infrared spectra of complex **1** before and after immersion in quinolone antibiotics; (**b**) PXRD diagram of complex **1** before and after immersion in quinolone antibiotics; (**c**) The liquid ultraviolet absorption spectra of complex **1**, LFX, OFX, and NFX; (**d**) The overlap between the emission spectra of complex **1** and the absorption spectra of LFX, OFX, and NFX.

To elucidate the recognition mechanism of quinolone antibiotics by complex **1**, we conducted comparative spectroscopic analyses. Firstly, in terms of structural stability, comparative FT-IR and PXRD studies of complex **1** before and after antibiotic exposure demonstrated no significant spectral changes (Figure 6a,b), indicating that no coordination bond was formed between the complex and quinolone antibiotics, and the coordination skeleton did not change after detection. Secondly, by comparing UV-vis absorption spectra of complex **1**, OFX, LFX, and NFX, substantial spectral overlap in the 200–300 nm region between complex **1** and all three quinolones (Figure 6c), but its fluorescence peak does not overlap with that of quinolone antibiotics, shown in Figure 6d, indicating that there is competitive absorption of excited state energy between complex **1** and quinolone antibiotics.

After that, it was found that the quantum yield of complex **1** increased with the addition of OFX, LFX, and NFX (shown in Table 2), indicating that energy transfer occurred in this process.

To better understand the molecular interactions between the fluorescent probe and the analyte, molecular docking technology was applied to predict the binding modes of complex **1** with OFX, LFX, and NFX, respectively. The binding energies of complex **1** to OFX, LFX, and NFX are −3.24, 2.77, and −4.21 kcal/mol. As shown in Figure S4a,d, the distances between the five-member ring and six-member ring of the ligand and conjugated rings of the OFX ring are from 4.1 Å to 4.6 Å, respectively, forming a weak π···π intermolecular interaction, which enhances the original π conjugation system and further strengthens the electron delocalization. Through electrostatic interactions, it stabilizes the excited state, thereby reducing non-radiative decay. This is consistent with the fluorescence enhancement phenomenon observed experimentally [28]. Similarly, multiple moderate π···π intermolecular interactions may occur between complex **1** and NFX (Figure S4c,f). In contrast to the interactions observed with OFX and NFX, a distinct hydrogen bond likely forms between the non-deprotonated COOH group of the ligand and an oxygen atom of LFX during sensing experiments (shown in Figure S4b,e). These extensive intermolecular interactions collectively contribute to the fluorescent probe’s high detection performance toward the three quinolone antibiotics.

## 4. Conclusions

In this study, we successfully designed and synthesized a novel terbium(III) complex, [Tb(HPPIPA)(PPIPA)(H_2_O)]ₙ, using 5-(pyrazol-1-yl)pyridine-2-carboxylic acid (H_2_PPIPA) as the organic ligand. This complex demonstrates exceptional performance as a turn-on fluorescent probe for the selective detection of quinolone antibiotics (OFX, LFX, and NFX), achieving ultra-low detection limits (8.0–27.9 nM). The practical applicability was confirmed through successful quantification of OFX and LFX in milk matrices, with excellent recovery rates (90.80–106.73%) and minimal interference from other antibiotics. Furthermore, we developed a reusable fluorescent agarose membrane incorporating the complex, which enables naked-eye, on-site detection of quinolones under UV light (254 nm) with reversible responsiveness over seven cycles. This work might provide an experimental foundation for the highly efficient, rapid, and visual detection of quinolone antibiotics in real samples.

## Figures and Tables

**Table 1:** The determination results of OFX and LFX in commercial milk (the concentration is expressed as the average value of three measurements).

Antibiotics	C (μM)	Founded (μM)	Recovery Rate (%)	RSD (%, n = 3)
**OFX**	7.5	7.42	98.93	3.1
	10	10.39	103.90	2.6
	15	16.01	106.73	1.2
**LFX**	7.5	7.28	97.07	2.0
	10	9.08	90.80	1.7
	15	15.73	104.87	0.9

**Table 2:** Quantum yield results of complex **1** before and after adding OFX, LFX and NFX.

Sample	Complex 1	Complex 1@OFX	Complex 1@LFX	Complex 1@NFX
QY (488 nm)	1.82%	22.56%	21.58%	
QY (545 nm)	5.53%			13.82%

## Data Availability

The original contributions presented in this study are included in the article/Supplementary Materials. Further inquiries can be directed to the corresponding author.

## Supplementary Materials

The following supporting information can be downloaded at https://www.mdpi.com/article/10.3390/polym17172277/s1. Figure S1: Experimental (red line) and simulation (black line) PXRD of complex **1**; Figure S2: TG analysis of complex **1**; Figure S3: Fluorescence intensity of complex **1** at pH = 2–13; Figure S4: Molecular docking of complex **1** with OFX, LFX and NFX, respectively; Figure S5: ^1^H NMR spectra of complex **1** in D_2_O; Table S1: Crystal date and structure refinement parameters for complex **1**; Table S2: Selected bond lengths (A) and angles (°) of complex; Table S3: Comparison of the performance of complex **1** with other fluorescent probes for the detection of quinolone antibiotics [1,15,16,29,30,31,32,33,34,35,36,37,38,39,40,41].
