# Supplementary material for: A Tb (Ⅲ) Coordination Polymer Based on 5-(2-(Pyrazole-1-yl) Pyridine-5-yl) Terephthalic Acid and Its Visual Detection of Quinolone Antibiotics
Source: Polymers (Basel). 2025 Aug 22;17(17):2277. doi: 10.3390/polym17172277 (PMC12431072; doi:10.3390/polym17172277)
Supplement: Supplementary file 1 [file polymers-17-02277-s001.zip › polymers-3799940-supplementary.pdf]

A Tb (Ⅲ) Coordination Polymer Based on 5-(2-(Pyrazole-1-yl) Pyridine-5-yl) Terephthalic Acid and Its Visual Detection of Quinolone Antibiotics

Ai Wang [1,2,*], Yichong Li [1], Wei Zhao [1] and Jia Liu [1]

[1] Key Laboratory of Chemical Biology and Molecular Engineering of the Education Ministry, Institute of Molecular Science, Shanxi University, Taiyuan 030006, China; liyichong1010@163.com (Y.L.); zw18435740421@163.com (W.Z.); liujia18635024993@163.com (J.L.)
[2] Key Laboratory of Materials for Energy Conversion and Storage of Shanxi Province, Shanxi University, Taiyuan 030006, China
* Correspondence: aiwang@sxu.edu.cn

Analyses of ^{1}H NMR spectra of complex **1** in D_2O
Figure S1 Experimental (red line) and simulation (black line) PXRD of complex **1**
Figure S2 TG analysis of complex **1**
Figure S3 Fluorescence intensity of complex **1** at pH = 2-13
Figure S4 Molecular docking of complex 1 with OFX, LFX and NFX, respectively
Figure S5 ^{1}H NMR spectra of complex **1** in D_2O.
Table S1 Crystal date and structure refinement parameters for complex **1**
Table S2 Selected bond lengths (A) and angles (°) of complex **1**
Table S3 Comparison of the performance of complex **1** with other fluorescent probes for the detection of quinolone antibiotics

Analyses of ^{1}H NMR spectra of complex 1 in D_2O

Chemical shift at 8.51 ppm are the two protons at the ortho-position relative to the nitrogen in the pyridine ring and one proton at the ortho-position relative to the uncoordinated -COOH of benzene ring. 8.46 ppm shift is assigned to the H atom at the ortho-position relative to the $\mu_{1,1}$-COO coordination mode. The chemical shift at 8.10 ppm corresponds to the ortho-hydrogens (H atoms adjacent) to the two nitrogen atoms on the chelated pyrazole ring. The peaks at 7.98, 7.88, and 7.61 ppm are assigned to the remaining hydrogen atoms of the pyridine ring and benzene ring, respectively. The resonance at 7.59 ppm is attributed to the ortho-hydrogens relative to the two nitrogen atoms on the uncoordinated pyrazole ring. The two hydrogen atoms at 6.66 ppm belong to the para-position carbons (C-H) of both pyrazole rings.

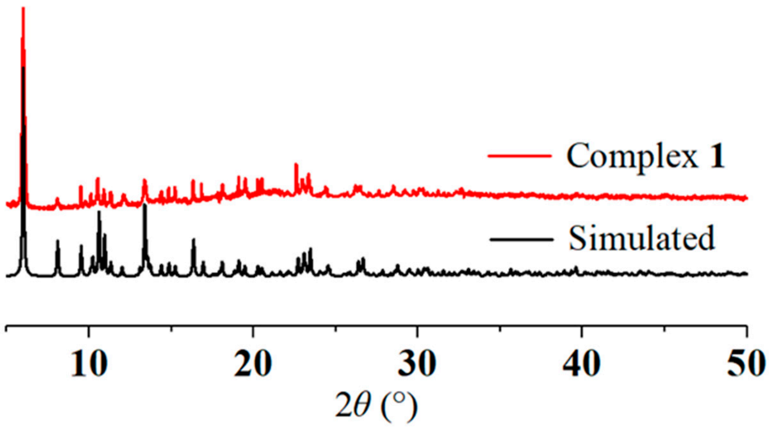

Figure S1 Experimental (red line) and simulation (black line) PXRD of complex **1**

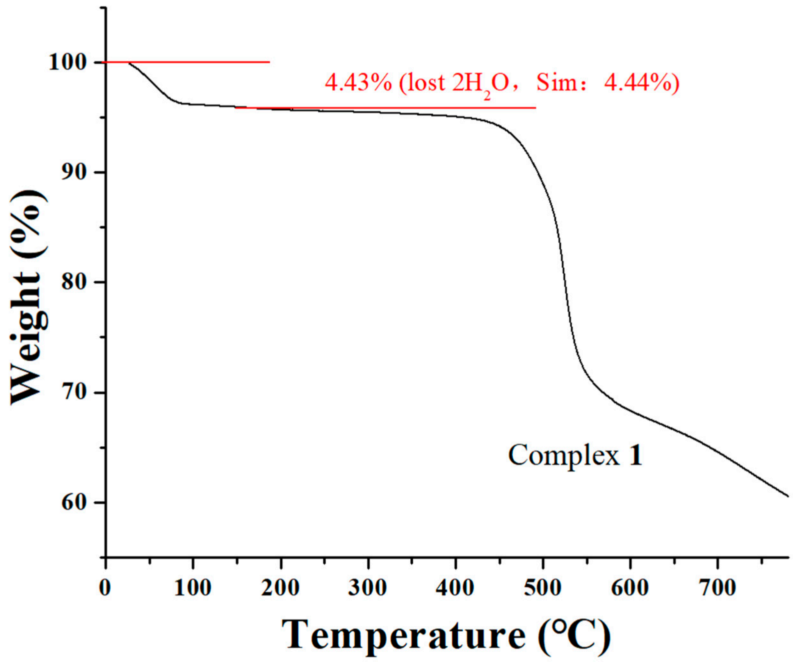

Figure S2 TG analysis of complex **1**

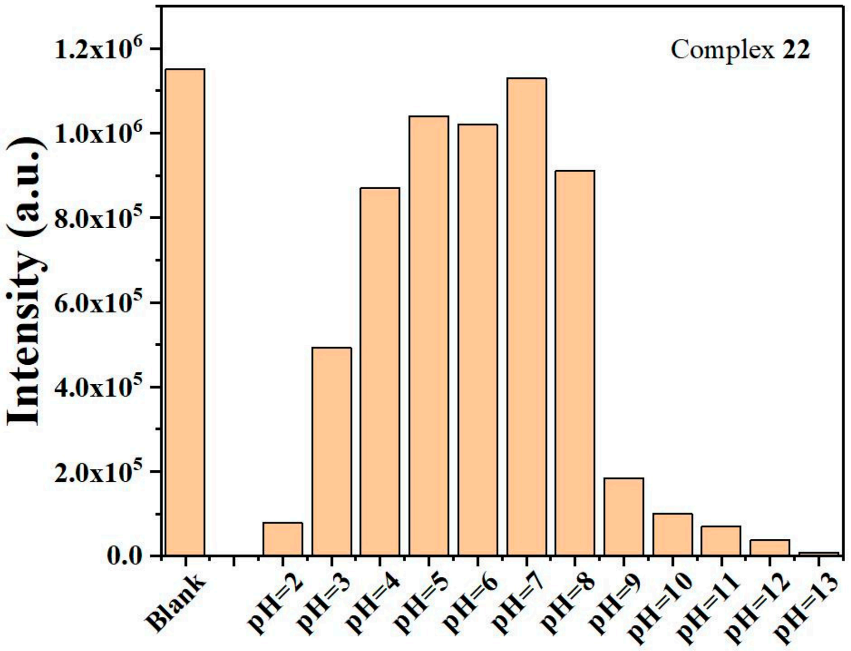

Figure S3 Fluorescence intensity of complex **1** at pH = 2-13

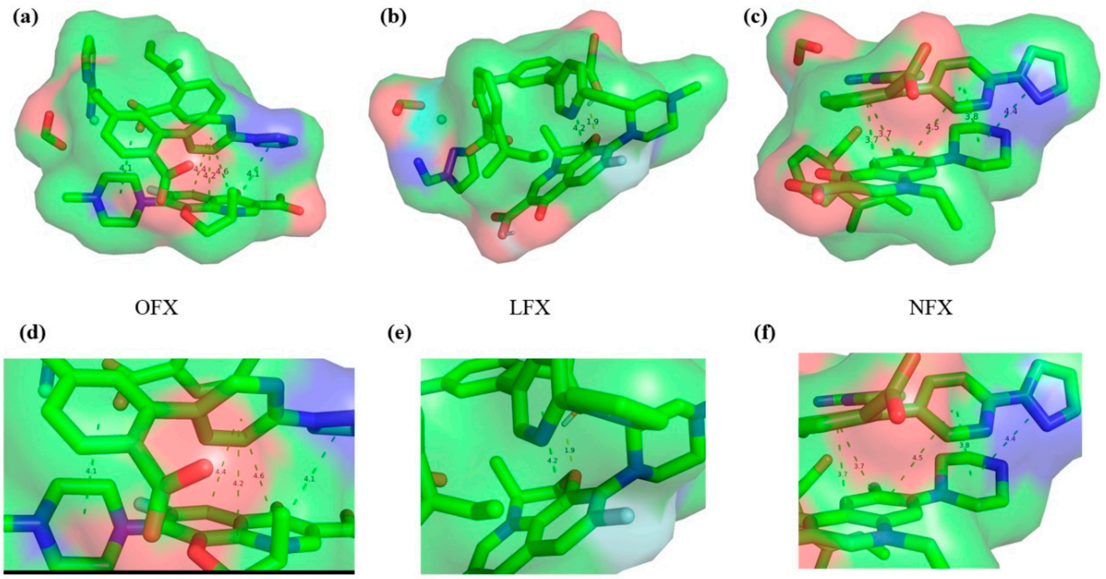

Figure S4 Molecular docking of complex **1** with OFX, LFX and NFX, respectively

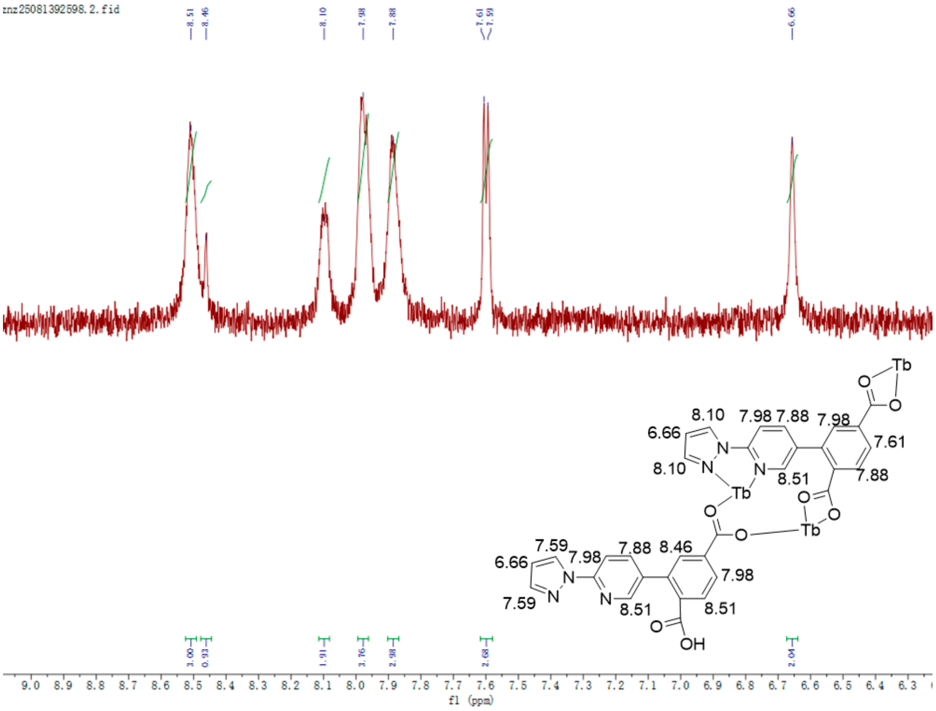

Figure S5 ^{1}H NMR spectra of complex **1** in D_2O

Table S1 Crystal date and structure refinement parameters for complex **1**

Complex	**1**
CCDC	2416360
Formula	$C_{32}H_{21}TbN_6O_9$
*M*r	810.45
Crystal system	Triclinic
Space group	*P*-1
Temperature (K)	273(2)
Wavelength (Å)	0.71073
a(Å)	9.5058(12)
b(Å)	11.4574(15)
c(Å)	15.517(2)
α(°)	91.065(5)
β(°)	107.217(4)
γ(°)	106.507(5)
V($Å^3$)	1538.0(3)
Z	2
Dx($Mg·m^{-3}$)	1.672
μ(mm^{-1})	2.36
F(000)	764
R_{int}	0.057
GOOF	1.07
R_1 [$F^2 > 2\sigma (F^2)$]	0.039
$wR_2(F^2)$	0.096
$\rho_{max,\ min}$($eÅ^{-3}$)	1.39, −1.61

Table S2 Selected bond lengths (A) and angles (°) of complex **1**

Complex **1**					
Bond	(Å)	Bond	(Å)	Bond	(Å)
Tb1—O2	2.333 (3)	Tb1—O7	2.414 (3)	Tb1—N6	2.526 (4)
Tb1—O5^{i}	2.336 (3)	Tb1—O6	2.458 (4)	Tb1—N4	2.572 (3)
Tb1—O1	2.383 (3)	Tb1—O8	2.481 (4)	Tb1—O5	2.633 (3)
Angle	(°)	Angle	(°)	Angle	(°)
O2—Tb1—O5^{i}	76.70 (11)	O5^{i}—Tb1—O6	126.26 (11)	O6—Tb1—O8	148.43 (12)
O2—Tb1—O1	136.62 (10)	O1—Tb1—O6	89.61 (13)	O2—Tb1—N6	135.33 (12)
O5^{i}—Tb1—O1	77.81 (11)	O7—Tb1—O6	137.77 (11)	O5^{i}—Tb1—N6	147.87 (12)
O2—Tb1—O7	75.37 (11)	O2—Tb1—O8	127.45 (11)	O1—Tb1—N6	74.69 (11)
O5^{i}—Tb1—O7	78.24 (11)	O5^{i}—Tb1—O8	81.77 (12)	O7—Tb1—N6	18.58 (13)
O1—Tb1—O7	131.81 (12)	O1—Tb1—O8	82.14 (11)	O6—Tb1—N6	70.9 (14)
O2—Tb1—O6	78.15 (13)	O7—Tb1—O8	53.41 (10)	O8—Tb1—N6	78.34 (14)
Symmetry codes: (i) $-x+1, -y+2, -z+1$; (ii) $-x+1, -y+1, -z+1$; (iii) $x-1, y, z$; (iv) $x+1, y, z$					

Sensor	Media	Analyte	LOD (M)	Ref.
$\{[Zn_2(L)(DCTP)_2]\cdot H_2O\}_n$	H_2O	LFX	2.47×10^{-9}	[1]
		NFX	1.39×10^{-8}	
GCDs@Eu-MOF		FXs	$4.3\text{-}6.5\times10^{-9}$	[2]
		**OFX**	$\mathbf{2.79\times10^{-8}}$	
**Complex 1**	$\mathbf{H_2O}$	**LFX**	$\mathbf{1.71\times10^{-8}}$	**This work**
		**NFX**	$\mathbf{8.01\times10^{-9}}$	
Tb-Eu-co-doped-MOFs	PBS	NFX	6.0×10^{-8}	[3]
S-CDs@Tb-MOF	H_2O	NFX	3.6×10^{-8}	[4]
		LFX	3.83×10^{-7}	
N, S-CDs@Tb-MOFs	H_2O	LFX	3.60×10^{-8}	[5]
EuTb@ME-IPA		NFX	4.80×10^{-8}	[6]
		CFX	3.70×10^{-8}	
NS-3	CH_3OH	OFX	3.40×10^{-8}	[7]
		NFX	4.30×10^{-8}	
Eu-2	DMSO	NFX	4.22×10^{-8}	[8]

	DMSO	CFX	6.25×10^{-9}	
Eu-1	DMSO	NFX	1.44×10^{-8}	
	DMSO	CFX	2.86×10^{-8}	
Mn^{2+}/Eu-MOFs	H_2O	PFX	1.50×10^{-8}	[9]
$Eu_{0.15}Tb_{2.85}(BDC)_{3.0}$	Urine	NFX	1.10×10^{-8}	[10]
RhB@Tb-dcpcpt	H_2O	NFX	1.70×10^{-7}	[11]
CS-CPDs	H_2O	LFX	2.00×10^{-7}	[12]
BUC-88	H_2O	NFX	5.30×10^{-7}	[13]
$[Eu_2L_{0.5}(IPA)_3]n$	H_2O	NFX	7.90×10^{-7}	[14]
Tb/Eu- BPDC	H_2O	NFX	9.26×10^{-7}	[15]
QDs	H_2O	GFLX	3.60×10^{-6}	[16]

Table S3 Comparison of the performance of complex **1** with other fluorescent probes for the detection of quinolone antibiotics

[1] Z. H. Gao, L. S. Fu, G. Y. Dong, *et al.* Two robust Zn-CPs as multiresponsive turn-on fluorescent sensors for the detection of five quinolone antibiotics. *J. Mol. Struct.*, **2024**, 1318, 139339.

[2] R. J. Tang, L. F. Li B. H. Liu, *et. al.* Ratiometric fluorescent sensor for sensitive visualization of full-spectrum fluoroquinolone antibiotic residues in the environment. *Microchem. J.*, **2025**, 212, 113260.

[3] R. Xie, P. Yang, J. Liu, X. Zou, Y. Tan, X. Wang, J. Tao, P. Zhao. Lanthanide-functionalized metal-organic frameworks based ratiometric fluorescent sensor array for identification and determination of antibiotics. *Talanta*, **2021**, 231, 122366.

[4] J. Chi, Y. Y. Song, L. Feng, *et al.* A ratiometric fluorescence sensor with different responsive modes based on carbon dots-embedded Tb-MOFs for the determination of norfloxacin and levofloxacin. *Talanta.*, **2024**, 280, 126763.原来的 2

[5] A. Sowndarya, T. Daniel Thangadurai, *et al.* Morphology-transforming AuNPs-based fluorescent probe for ultra-low sensitive detection of levofloxacin in urine samples at pH 7.0 through excimer formation. *J. Mol. Liq.*, **2024**, 407, 125156.

[6] Y. W. Ye, T. T. Wu, X. T. Jiang. J. X. Cao, X. Ling, Q. S. Mei, H. Chen, D. M. Han, J. -J. Xu, Y. Z. Shen. Portable Smartphone-Based QDs for the Visual Onsite Monitoring of Fluoroquinolone Antibiotics in Actual Food and Environmental Samples. *ACS Appl. Mater.*

Interfaces, **2020**, 12, 14552−14562.

[7] C. J. Lin, H. H. Wang, *et al*. A [Co5] cluster-based organic framework as fluorescent detection platform toward quinolone antibiotics. *Appl. Organomet. Chem*., **2024**, 38, 7.

[8] T. Xiao, D. -D.Yang, Y. -S. Shi, H. -W. Zheng, Z. -G. Xia, and X. -J. Zheng. Hydrazone-Based Europium(III) Complexes: Mechanochromic Luminescenceand Turn-On Fluorescence Detection of Quinolone Antibioticsin Human Urine. *Cryst. Growth Des*. **2023**, 23, 5957−5964.

[9] C. -Y. Wang, C. -C. Wang, X. -W. Zhang, *et al*. A new Eu-MOF for ratiometrically fluorescent detection toward quinolone antibiotics and selective detection toward tetracycline antibiotics. *Chin. Chem. Lett*., **2022**, 33,1353–1357.

[10] D. H Wang, H. Kang, X. R. Wang, W. Zhou. Fluorescence turn off-on continuous response of dual lanthanide metal organic frameworks for selective detecting fluoroquinolone antibiotics.

[11] M. Yu, Y. Xie, X. Wang, Y. X. Li, G. Li. Highly water-stable Dye@Ln-MOFs for sensitive and selective detection toward antibiotics in water. *ACS Appl Mater Inter*, **2019**. 11(23), 21201–21210.

[12] J. S. Lin, S. L. Yang, Y. J. Wang, *et al*. Sensitive detection of levofloxacin and copper (II) based on fluorescence “turn on-off” of biomass carbonized polymer dots. *J. Ind. Eng. Chem*, **2022**, 114, 288-296.

[13] D. D. Yang, Y. S. Shi, T. Xiao, *et al*. Three-Dimensional Viologen-Based Lanthanide-Organic Frameworks: Photochromism and Fluorescence Detection of Quinolone Antibiotics. *Inorg. Chem*., **2023**, 62, 15, 6084–6091.

[14] Y. J. Guo, L. F. Li, S. H. Xu, M. F. Zhang, *et al*. Ion coordination and chelation in Eu-MOFs matrices: Ultrafast fluorescence visual quantification monitoring of antibiotic residues. *Talanta.,* **2024***,* 278, 126549.

[15] M. Xia, W. C. Ma, L. N. Zhao, H. B. Liu. Highly Selective and Sensitive Detection of Quinolone Antibiotics Using Lanthanide Metal–Organic Framework- Based White- Light Materials. *Luminescence*, **2025**, 40, e70227.

[16] S. Meng, B. F. Mu, S. Mao, Z. Li. Dual-lanthanide functionalized hydrogen-bonded organic frameworks for fluorescent detection of quinolone antibiotics in multi-media. *J. Hazard. Mater.*, **2025**, 496, 139335.
